# Receptor interaction profiles of 4-alkoxy-2,6-dimethoxyphenethylamines (Ψ derivatives) and related amphetamines

**DOI:** 10.3389/fphar.2025.1703480

**Published:** 2025-11-20

**Authors:** Karolina E. Kolaczynska, Daniel Trachsel, Marius C. Hoener, Matthias E. Liechti, Dino Luethi

**Affiliations:** 1 Division of Clinical Pharmacology and Toxicology, Department of Biomedicine, University Hospital Basel and University of Basel, Basel, Switzerland; 2 Department of Pharmaceutical Sciences, University Hospital Basel and University of Basel, Basel, Switzerland; 3 ReseaChem GmbH, Kehrsatz, Switzerland; 4 Neuroscience Research, pRED, Roche Innovation Center Basel, F. Hoffmann-La Roche Ltd., Basel, Switzerland

**Keywords:** psychedelic, phenethylamine, serotonin, receptor, pseudo derivatives, fluorination

## Abstract

**Background:**

4-substituted 2,6-dimethoxyphenethylamines and the corresponding amphetamines (so-called pseudo [Ψ] derivatives) are a hitherto mostly unexplored group of psychedelics. Still, preliminary investigations indicate that these derivatives are promising and potent psychedelics in humans. In this study, we examined the monoamine receptor and transporter interaction properties of several 4-alkyloxy-2,6-dimethoxyphenethylamines and amphetamines with varying structural modifications at the 4-alkyloxy position and compared them to structural analogs with 3,4,5- and 2,4,5-substitution patterns.

**Methods:**

Binding affinities were assessed at human serotonergic 5-HT_1A_, 5-HT_2A_, and 5-HT_2C_ receptors, adrenergic α_1A_ and α_2A_ receptors, dopaminergic D_2_ receptor, rat and mouse trace-amine associated receptor 1 (TAAR1), and human monoamine transporters. Moreover, the Ψ derivatives were examined for their activation potency at human 5-HT_2A_ and 5-HT_2B_ receptors and at human TAAR1.

**Results:**

The tested derivatives displayed moderate to high affinity and activity at the h5-HT_2A_ receptor (*K*_i_ = 8–1,600 nM; EC_50_ = 32–3,400 nM). All derivatives were partial agonists at the receptor (activation efficacy ≤84%). Moreover, the phenethylamine derivatives bound to the h5-HT_1A_ (*K*_i_ = 710–4,440 nM) and h5-HT_2C_ (*K*_i_ = 110–3,500 nM) receptors with moderate affinity, whereas the amphetamine derivatives showed weak h5-HT_1A_ affinities (*K*_i_ ≥ 5,100 nM) and comparably lower h5-HT_2C_ receptor affinities (*K*_i_ = 270–10,000 nM). Within the remaining receptors investigated, some of the Ψ derivatives showed significant interactions with the human (EC_50_ ≥ 34 nM), rat (*K*_i_ ≥ 1.6 nM), and mouse (*K*_i_ ≥ 120 nM) TAAR1, the hα_1A_ adrenoceptor (*K*_i_ ≥ 670 nM) and the hα_2A_ adrenoceptor (*K*_i_ ≥ 280 nM).

**Conclusion:**

The Ψ derivatives mainly interacted with the 5-HT_2A_ receptor, the primary target for psychedelics, as well as with the 5-HT_2C_ receptor. The same 4-alkyloxy modification pattern on the related 2,4,5-trisubstituted derivatives exhibited generally slightly more potent 5-HT_2A_ receptor binding and activation, whereas 3,4,5-trisubstituted derivatives interacted with lower potency; in humans, 2,4,6-trisubstituted derivatives may thus be less potent compared to their 2,4,5-trisubsititued counterparts but more potent compared to their 3,4,5-trisubsititued counterparts.

## 1 Introduction

Central nervous system processes like sexual activity, sleep, appetite, or memory are all, to different degrees, governed by their interactions with the serotonergic system (Berger et al., 2009). Serotonin (5-hydroxytryptamine, 5-HT) and its various receptor subtypes are widespread in the brain and spinal cord; thus, a dysfunction in their regulation may lead to psychiatric conditions like anxiety or depression (Berger et al., 2009; Rapport et al., 1948). The 5-HT_2A_ and 5-HT_2C_ receptor subtypes are pharmacological targets for natural and synthetic psychedelics like psilocin (active metabolite of psilocybin) and lysergic acid diethylamide (LSD) (Glennon et al., 1992; Vollenweider et al., 1998; Chambers et al., 2002; Preller et al., 2017; Nichols, 2016). While the 5-HT_2A_ receptor’s involvement in the production of psychedelic effects is well understood (Vollenweider et al., 1998; Nichols, 2016; Vargas et al., 2023; Nichols, 2004), the role of the 5-HT_2C_ receptor remains more enigmatic. For instance, 5-HT_2C_ receptor antagonism does not alter 4-iodo-2,5-dimethoxyamphetamine (DOI)-induced head-twitch in mice, whereas the head-twitch is noncompetitively inhibited by 5-HT_2C_ receptor agonism (Fantegrossi et al., 2010). For agonists, selective binding to either one of the receptor subtypes remains difficult due to their high degree of sequence homology in ligand binding sites (Boess and Martin, 1994; Trachsel et al., 2009).

The three main isoforms of the 5-HT_2_ receptor subtype (i.e., 5-HT_2A_, 5-HT_2B_, and 5-HT_2C_) are pharmacological targets for several antipsychotic medications (Roth, 2011). Initially, it has been difficult to establish the role of each 5-HT receptor subtype in disease, as most ligands targeting the 5-HT_2_ receptors are nonselective (Glennon et al., 1992). However, a surge of preferential or even selective ligands for each 5-HT_2_ receptor subtype has since been identified, at least within antagonists. Substituted phenethylamines like the psychedelic 4-bromo-2,5-dimethoxyamphetamine (DOB) belong to full or partial agonistic ligands that exhibit high affinity, binding preferentially to the 5-HT_2_ receptor family but express relatively low selectivity between the three receptor isoforms (Glennon et al., 1992; Chambers et al., 2002; Monte et al., 1996). However, a few ligands with agonistic binding properties display a remarkable preferential 5-HT_2A_ over 5-HT_2C_ binding profile (Hansen et al., 2014; Juncosa et al., 2013). Likewise, selective 5-HT_2C_ agonists have been described (Cheng et al., 2015).

So far, a considerable number of phenethylamines have been characterized in terms of their 5-HT_2_ receptor interactions and their potential to induce psychoactive effects in humans (Luethi et al., 2019a; Luethi et al., 2018; Rickli et al., 2015; Eshleman et al., 2018; Trac and hsel, 2003; Trachsel et al., 2013; Glennon and Young, 1982; Barfknecht and Nichols, 1975; Aldous et al., 1974; Kolaczynska et al., 2019; Kolaczynska et al., 2022), and psychedelic phenethylamines are the biggest family of psychedelics investigated. Derived from 3,4,5-trimethoxyphenethylamine (mescaline), the archetypal and first psychedelic compound available in chemically pure form to man (Heffter, 1898), these derivatives can, due to their structure-activity relationships (SARs), be further segregated into three subgroups, largely based on their aryl substitution pattern; the 2,4,5-trisubstituted (2C and DOx derivatives), the 2,4,6-trisubstituted Ψ derivatives (Ψ-2C and Ψ-DOx series), and the 3,4,5-trisubstituted (scalines and 3C-scalines) compounds. Among the most intensely investigated compounds, the 2,4,5-series, it has been shown that, for agonistic binding properties, the aryl moiety needs to be 2,5-dimethoxy substituted, paired with small lipophilic 4-substituents such as methyl, ethyl, halogen, or alkylthio (Nichols, 2016; Nichols, 2004; Luethi and Liechti, 2018). Within the 3,4,5-series, a 3,5-dimethoxy pattern paired with a small lipophilic 4-substituent such as alkoxy, alkylthio (Kolaczynska et al., 2022; Shulgin and Shulgin, 1991), alkyl, alkenyl and alkynyl as well as fluorinated alkyl (Trachsel et al., 2023) is leading to compounds with the highest agonistic binding as well as psychedelic properties (Trachsel et al., 2013; Kolaczynska et al., 2022; Shulgin and Shulgin, 1991; Jacob and Shulgin, 1984; Jacob and Shulgin, 1981; Braun et al., 1978). Much less is known about the SAR of the 2,4,6-series, albeit, from the few known positional analogs some conclusions could already be drawn [reviewed in Trachsel et al. (2013)]. In this study, we focused on the largely unexplored 2,4,6-trisubstituted phenethylamines and their amphetamine counterparts. Although 2,4,6-trimethoxyphenethylamine and 2,4,6-triethoxyphenethylamine were synthesized in the 1950s (Benington et al., 1954), 2,4,6-substituted phenethylamines remain among the least explored psychedelic derivatives. This likely reflects the synthetic difficulty of introducing a substituent at the 4-position within a 2,4,6-substitution pattern (Shulgin and Shulgin, 1991; Trachsel, 2012). Phenethylamines are typically prepared by first establishing the aromatic substitution pattern, followed by side-chain introduction. While symmetrical derivatives are straightforward to obtain, regioselectivity complicates the synthesis of unsymmetrical 2,4,6-substituted analogs. For instance, electrophilic substitution on 2,6-dimethoxybenzaldehyde favors the 3-position, hindering access to 4-substituted derivatives. 3,5-Dimethoxy-4-hydroxybenzaldehyde, the key starting material for scalines and 3C-scalines, was already reported in 1948 (Pearl, 1948); in contrast, the preparation of 2,6-dimethoxy-4-hydroxybenzaldehyde, an essential precursor for 4-alkoxy-2,6-dimethoxy compounds, was only achieved decades later. Consequently, the relative ease of accessing 4-substituted 2,5-dimethoxyphenethylamines has driven much of the exploration within the 2,4,5-substitution series. 2,4,6-Trisubstituted derivatives are generally referred to as the pseudo series, with the greek letter psi (Ψ) as the prefix; by definition, Ψ derivatives generally contain the same aryl substituents as the compounds from the 2,4,5-trisubstituted series, with the 5-methoxy group being shifted to the 6-position. Thus, very few psychedelics and 5-HT_2A_ ligands derive from the pseudo series, bearing 2,6-dimethoxy substituents and a small, lipophilic group at the 4-position (Trachsel et al., 2013; Shulgin and Shulgin, 1991).

**FIGURE 1:**
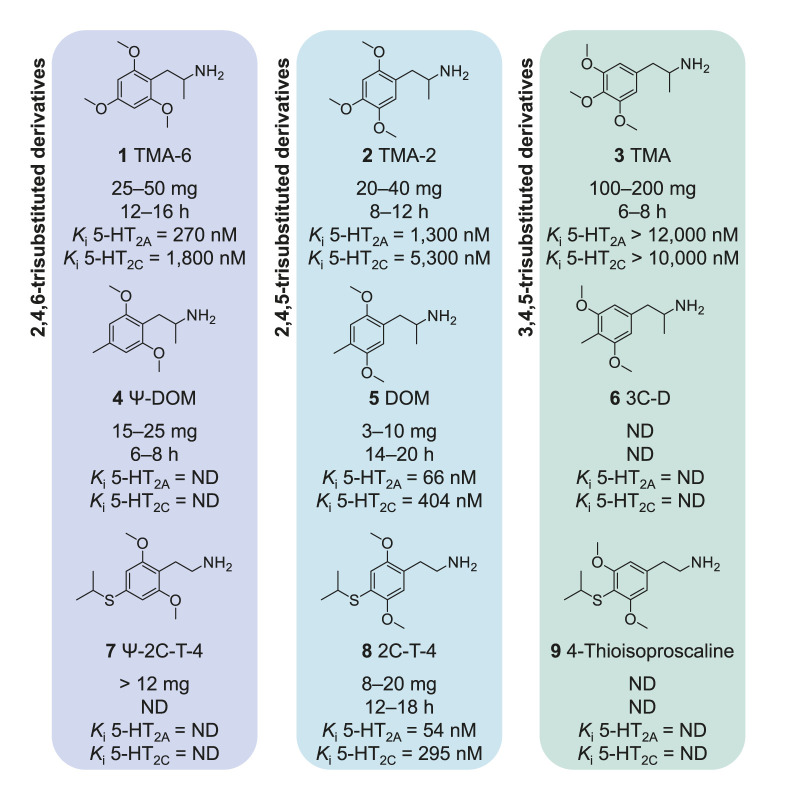
Initially explored Ψ derivatives in comparison to their 2,4,5- and 3,4,5-trisubstituted counterparts. Affinity for the human 5-HT_2A_ and 5-HT_2C_ receptors was assessed in this study or previously using identical assays (Luethi et al., 2018; Kolaczynska et al., 2019; Kolaczynska et al., 2022).

Some of the first few Ψ derivatives explored were 2,4,6-trimethoxyamphetamine (TMA-6; **1**), 2,6-dimethoxy-4-methylamphetamine (Ψ-DOM or Z-7; **4**), and 2,6-dimethoxy-4-isopropylthiophenethylamine (Ψ-2C-T-4; **7**) (Shulgin and Shulgin, 1991) (Figure 1). In humans, the first two compounds exhibit relatively potent psychoactive effects, albeit slightly less potent when compared to their 2,4,5-trisubstituted counterparts DOM (**5**) and TMA-2 (**2**). For the third compound, Ψ-2C-T-4 (**8**), an active dose has not yet been determined, since the compound proved to be inactive at the highest dose evaluated (12 mg) in an anecdotal report (Shulgin and Shulgin, 1991). From two other compounds, namely Ψ-2C-O-35 (**18**; 17 mg dose and about 18 h effect duration) and Ψ-DODFMO (**26**; 2 × 5 mg dose, about 20 h effect duration), some psychoactivity has been reported, although the effects of these compounds has not yet been fully explored (Trachsel et al., 2013). Their non-pseudo 2,4,5-analogs 2C-O-35 and DODFMO have been prepared but remain mostly unexplored (unpublished data). For the few pseudo compounds investigated *in vitro*, it has been shown that these derivatives bind with a somewhat lower affinity to the 5-HT_2A_ receptor (Chambers et al., 2002; Parker et al., 2008) and they have been found to be less potent in drug discrimination studies in rats (Glennon et al., 1981; Nichols et al., 1991), which is in line with the anecdotal human data. However, for the comparators TMA-2 (**2**) and TMA-6 (**3**), a nearly identical potency has been observed both in human and in animal. Thus, these preliminary investigations and the fact that many active 2,4,5-trisubstituted derivatives have largely unexplored 2,4,6-trisubstituted counterparts suggest that the Ψ derivatives are an exciting and promising series of potential psychedelic derivatives to investigate in more depth (Shulgin and Shulgin, 1991).

**FIGURE 2:**
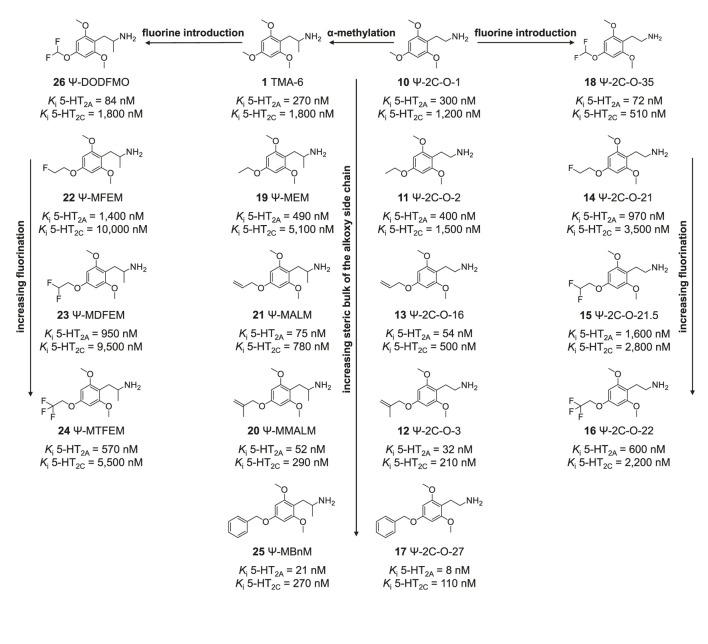
Chemical structures of the studied Ψ derivatives. Overall, increasing fluorination and increasing steric bulk of the alkoxy side chain of the studied phenethylamine derivatives (right) and their amphetamine counterparts (left) increased the affinity for the human 5-HT_2A_ and 5-HT_2C_ receptors.

**FIGURE 3:**
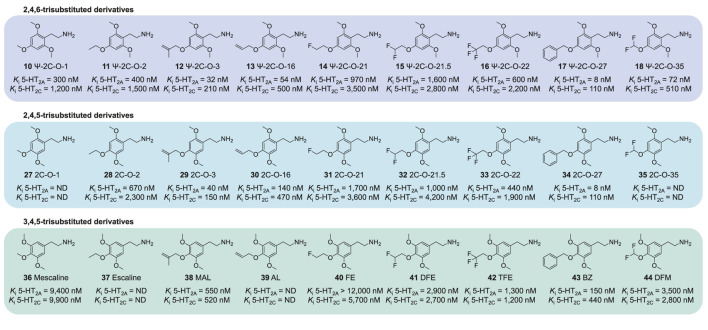
Comparison of phenethylamine-type Ψ derivatives and their 2,4,5- and 3,4,5-trisubstituted counterparts. Binding affinity data for the 2,4,5- and 3,4,5-trisubstituted derivatives were assessed in previous studies (Kolaczynska et al., 2019; Kolaczynska et al., 2022).

**FIGURE 4:**
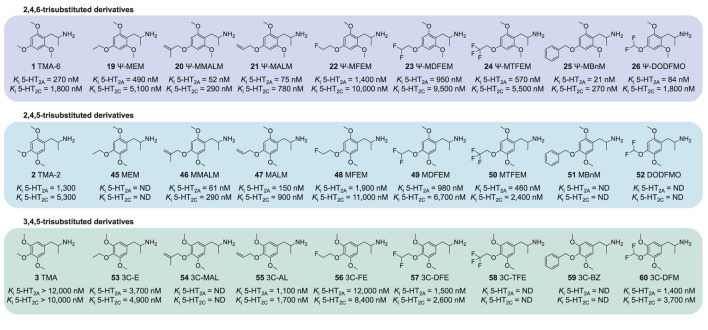
Comparison of amphetamine-type Ψ derivatives and their 2,4,5- and 3,4,5-trisubstituted counterparts. Binding affinity data for the 2,4,5- and 3,4,5-trisubstituted derivatives were assessed in previous studies (Kolaczynska et al., 2019; Kolaczynska et al., 2022).

We have previously examined a series of 4-alkoxy-2,5-dimethoxyphenethylamines (2C-O series) and their amphetamine counterparts (MRM series) *in vitro* (Kolaczynska et al., 2019). In that study, we found that the most active compounds at the 5-HT_2A_ receptor in terms of receptor affinity as well as activation potency and efficacy possessed 4-allyloxy and 4-methallyloxy moieties (e.g., MALM and MMALM). In comparison, 4-alkoxy-3,5-dimethoxyphenethylamines and amphetamines were less potent (Kolaczynska et al., 2022). In the current study, we determined the monoamine receptor and transporter binding and activation properties of several Ψ derivatives with varying structural modifications at the 4-alkyloxy position (Figure 2; structures **1, 10–26**). We compared the observed effects of these structural modifications to previously published data on 2C-O derivatives (Figure 3, structures **27–35**) and scalines (Figure 3, structures **36–44**), as well as their α-methyl counterparts, MRM derivatives (Figure 4, structures **2, 45–52**) and 3C-scalines (Figure 4, structures **3, 53–60**), respectively.

## 2 Materials and methods

### 2.1 Drugs

The 2,4,6-trisubstituted phenethylamines Ψ-2C-O-1, Ψ-2C-O-2, Ψ-2C-O-3, Ψ-2C-O-16, Ψ-2C-O-21, Ψ-2C-O-21.5, and Ψ-2C-O-22, Ψ-2C-O-27, Ψ-2C-O-35 (Figure 2, structures **10**–**18**) and the 2,4,6-trisubstitued amphetamines TMA-6, Ψ-MEM, Ψ-MMALM, Ψ-MALM, Ψ-MFEM, Ψ-MDFEM, and Ψ-MTFEM Ψ-MBnM, Ψ-DODFMO (Figure 2, structures **1, 19–26**) were synthesized and prepared as hydrochloride salts with a purity of >98% by ReseaChem (Kehrsatz, Switzerland) [^3^H]norepinephrine (13.1 Ci/mmol) and [^3^H]dopamine (30.0 Ci/mmol) were acquired from PerkinElmer (Schwerzenbach, Switzerland), while [^3^H]5-HT (80.0 Ci/mmol) was purchased from Anawa (Zürich, Switzerland).

### 2.2 Radioligand receptor and transporter binding

The binding affinity (*K*_i_) at numerous receptors and transporters was determined using radioligand displacement assays previously described by Luethi et al. (2018). Cell membrane preparations were derived from human embryonic kidney (HEK) 293 cells (serotonergic 5-HT_1A_, 5-HT_2A_, and 5-HT_2C_ receptors, trace amine-associated receptor 1 [TAAR1], dopaminergic D_2_ receptor, and monoamine transporters), Chinese hamster lung cells (α_2A_ adrenergic receptor), and Chinese hamster ovary cells (α_1A_ adrenergic receptor). In summary, the various cell lines were all transfected with their respective receptor or transporter targets (human genes and additionally mouse and rat genes for TAAR1). Subsequently, cell membrane preparations were prepared and incubated with selective radiolabeled ligands at concentrations equal to the dissociation constant [*K*_d_]). Displacement of the radioligands by the test substances was then assessed. Specific binding was determined as the difference between the total binding and nonspecific binding (assessed in the presence of respective receptor competitors in excess). The following radioligands and competitors, respectively, were used: 0.90 nM [^3^H]8-hydroxy-2-(dipropylamino)tetralin (8-OH-DPAT) and 10 μM pindolol (5-HT_1A_ receptor), 0.40 nM [^3^H]ketanserin and 10 μM spiperone (5-HT_2A_ receptor), 1.4 nM [^3^H]mesulergine and 10 μM mianserin (5-HT_2C_ receptor), 3.5 nM or 2.4 nM [^3^H]RO5166017 and 10 μM RO5166017 (rat and mouse TAAR1, respectively), 0.11 nM [^3^H]prazosin and 10 μM chlorpromazine (α_1_ adrenergic receptor), 2 nM [^3^H]rauwolscine and 10 μM phentolamine (α_2_ adrenergic receptor), 1.2 nM [^3^H]spiperone and 10 μM spiperone (dopaminergic D_2_ receptor), 2.9 nM *N*-methyl-[^3^H]nisoxetine and 10 μM indatraline (NET), 1.5 nM [^3^H]citalopram and 10 μM indatraline (SERT), 3.3 nM [^3^H]WIN35,428 and 10 μM indatraline (DAT). Binding affinities were determined using the Cheng-Prusoff equation. High affinity binding was defined as *K*_i_ < 50 nM, moderate affinity binding as *K*_i_ = 50–1,000 nM, and low affinity binding as *K*_i_ > 1,000 nM.

### 2.3 Activation potency and efficacy at the 5-HT_2A_ receptor

The activation potency and efficacy at the 5-HT_2A_ receptor were determined as previously described by Luethi et al. (2018). In short, mouse embryonic fibroblasts (NIH-3T3 cells) stably transfected with the human 5-HT_2A_ receptor were seeded in poly-D-lysine-coated 96 well plates at a density of 70,000 cells per 100 μL. Next, the cells were incubated in HEPES-Hank’s Balanced Salt Solution (HBSS) buffer (Gibco) for 1 h at 37 °C; the cells were then incubated with 100 μL of dye solution (fluorescence imaging plate reader [FLIPR] calcium 5 assay kit; Molecular Devices, Sunnyvale, CA, United States) per well for 1 h at 37 °C. The plate was then inserted into the FLIPR and 25 μL of the test compounds diluted in HEPES-HBSS buffer containing 250 mM probenecid were added online. Increase of the fluorescence signal was measured and the activation potency (EC_50_) was calculated from the dose-response curves using nonlinear regression. The activation efficacy was calculated relative to 5-HT activity (defined as 100% activity).

### 2.4 Activation potency and efficacy at the 5-HT_2B_ receptor

The activation potency and efficacy at the 5-HT_2B_ receptor were determined as previously described by Luethi et al. (2018). In summary, HEK 293 cells stably transfected with the human 5-HT_2B_ receptor were seeded in poly-D-lysine-coated 96 well plates at a density of 50,000 cells per well overnight at 37 °C in high glucose Dulbecco’s modified Eagle’s medium (DMEM; Invitrogen, Zug, Switzerland) supplemented with 10% fetal calf serum (non-dialyzed and heat-inactivated), 250 mg/L Geneticin, and 100 U/mL penicillin, and 100 μg/mL streptomycin (Gibco). The growth medium was removed by snap inversion and the cells were exposed to 100 μL calcium indication Fluo-4-solution (Molecular Probes, Eugene, OR, United States) for 45 min at 31 °C. Thereafter, the Fluo-4-solution was removed once again by snap inversion and was replaced with fresh 100 μL Fluo-4-solution. Next, the cells were washed with HBSS and 20 mM HEPES using the EMBLA cell washer and then exposed to the assay buffer (100 μL per well). Afterwards, 25 μL of test compounds diluted in assay buffer were added to the plate online. Dose–response curves were determined using nonlinear regression and EC_50_ values were calculated. The activation efficacy was calculated relative to the 5-HT activity (defined as 100%).

### 2.5 Activation potency at human TAAR1

The activation potency at the human TAAR1 was determined as previously described by Luethi et al. (2018). In summary, recombinant HEK 293 cells stably transfected with the human TAAR1 were grown in culture flasks composed of 30 mL high glucose DMEM with 10% heat inactivated fetal calf serum, 500 μg/L Geneticin, and 500 μg/L hygromycin B at 37 °C and 5% CO_2_. The cells were grown to 80%–90% confluency before collection. The cells were washed with phosphate-buffer saline (PBS) and 5 mL of trypsin/EDTA solution were added for 5 min at 37 °C to detach the cells from the flask. Then, 45 mL of fresh medium were added to the flask and the total mixture was put into a falcon tube, which was centrifuged for 3 min at 900 rpm at room temperature. The supernatant was aspirated, and the remaining cell pellet was resuspended in fresh medium to a concentration of 5 × 10^5^ cells per mL. Next, 100 μL of the cell mixture was transferred to a 96 well plate (BIOCOAT 6640, Becton Dickinson, Allschwil, Switzerland) and incubated for 20 h at 37 °C. For the cAMP assay, the medium was removed and replaced with 50 μL PBS lacking calcium or magnesium ions. The PBS was extracted *via* snap inversion and the plate was gently tapped on tissue to remove any remaining PBS solution. The plate was then incubated with Krebs-Ringer Bicarbonate buffer (90 μL/well; KRB, Sigma-Aldrich) containing 1 mM IBMX for 1 h at 37 °C and 5% CO_2_/95% air. Test compounds were investigated in duplicate at various concentrations ranging from 300 pM to 30 μM. A cAMP standard curve (concentration range of 0.13 nM–10 μM) was included on each 96 well plate. Additionally, a reference plate was used containing RO5256390, β-phenylethylamine and p-tyramine. Cells were incubated with either 30 μL of compound solution, β-phenylethylamine (which represented the maximal response) or a basal control in PBS (supplemented with 1 mM IBMX) for 40 min at 37 °C. Thereafter, the cells were incubated with 50 μL of 3× detection mix solution (consisting of Ru-cAMP Alexa700 anti-cAMP antibody and lysis buffer) for 40 min at 37 °C, while covered with black lids and under forceful shaking. The fluorescence (456 nm excitation wavelength; 630 and 700 nm emission wavelengths) was measured using a NanoScan reader (Innovate Optische Messtechnik, Berlin, Germany) and the FRET signal was calculated using the following equation: FRET (700 nm) – P × FRET (630 nm). P was equal to Ru (700 nm)/Ru (630 nm).

### 2.6 Monoamine uptake transporter inhibition

The monoamine uptake transporter inhibition potential of the test drugs at a concentration of 10 μM was determined in HEK 293 cells stably transfected with the respective transporters (Luethi et al., 2019b). The cells were grown to a confluency of 70%–90% in DMEM medium supplemented with 10% fetal calf serum and 250 μg/mL Geneticin (Gibco, Zug, Switzerland). Thereafter, the cells were detached with trypsin and resuspended in Krebs-Ringer bicarbonate buffer (Sigma-Aldrich) at a density of 3 × 10^6^ cells per mL. For [^3^H]DA uptake experiments, the buffer was additionally supplemented with 0.2 mg/mL ascorbic acid (Sigma-Aldrich). In brief, 100 μL of cell suspension per well was exposed to 25 μL of test drugs, control (0.1% DMSO), or 10 μM transporter-specific inhibitors (nisoxetine for NET, fluoxetine for SERT, and mazindol for DAT) dissolved in buffer for 10 min in a round-bottom 96 well plate. The mixture was placed on a rotary shaker at 450 rpm at room temperature. Uptake was initiated by the addition of 50 μL of radiolabeled neurotransmitter dissolved in buffer at a final concentration of 5 nM. After 10 min, 100 μL of the cell mixture was transferred into microcentrifuge tubes containing 50 μL of 3 M KOH and 200 μL silicon oil (1:1 mixture of silicon oil types AR 20 and AR 200; Sigma-Aldrich) and centrifugated for 3 min at 13,200 rpm in order to separate the cells from the uptake buffer. Directly after centrifugation, the tubes were frozen in liquid nitrogen. Afterwards, the cell pellets were cut off and transferred into scintillation vials (PerkinElmer) containing 0.5 mL of lysis buffer (1% NP-40, 5 mM EDTA, 0.05 M Tris-HCL, 50 mM NaCl in H_2_O) and placed on a shaker for 1 h at 700 rpm. Next, 3.5 mL of scintillation fluid (Ultima Gold, PerkinElmer) were added to each vial. Radioactivity was measured on a liquid scintillation counter (Packard 1900 TR Tri-Carb Liquid Scintillation Counter; Packard Instrument Company). Nonspecific uptake, which was determined in the presence of the selective inhibitors, was subtracted from the total uptake. The data from at least three independent experiments were analyzed and compared to controls using one-way ANOVA followed by Dunnett’s multiple-comparison test.

## 3 Results

### 3.1 Binding and activation of serotonin receptors

#### 3.1.1 5-HT_1A_ receptor

5-HT_1A_ receptor affinities are shown in Table 1. Ψ-2C-O-1 (**10**), Ψ-2C-O-2 (**11**), and Ψ-2C-O-16 (**13**) exhibited submicromolar binding at the 5-HT_1A_ receptor (*K*_i_ = 710–960 nM). All remaining phenethylamine derivatives (**12, 14**–**38**) bound to the receptor in the low micromolar range (*K*_i_ = 1,010–4,440 nM). In contrast, the amphetamine derivatives (**1, 19**–**26**) did not bind to the receptor in the examined concentration range (*K*_i_ > 5,600 nM) with the exception of Ψ-DODFMO (**26**), which showed low micromolar affinity (*K*_i_ = 5,100 nM).

**TABLE 1:** Serotonin receptor binding affinities and activation potencies of 4-alkoxy-substituted 2,6-dimethoxyphenethylamines and amphetamines.

		h5-HT_1A_	h5-HT_2A_			h5-HT_2B_		h5-HT_2C_	Selectivity (binding ratios)	
		Receptor binding	Receptor binding	Activation potency	Activation efficacy	Activation potency	Activation efficacy	Receptor binding	5-HT_2A_/5-HT_1A_	5-HT_2A_/5-HT_2C_
		*K*_i_ ± SD [nM]	*K*_i_ ± SD [nM]	EC_50_ ± SD [nM]	E_max_ ± SD [%]	EC_50_ ± SD [nM]	E_max_ ± SD [%]	*K*_i_ ± SD [nM]		
		[^3^H]8-OH-DPAT	[^3^H] ketanserin					[^3^H] mesulergine		
4-alkoxy-substituted 2,6-dimethoxyphenethylamines										
**10**	Ψ-2C-O-1	960 ± 90	300 ± 40	110 ± 20	84 ± 13	330 ± 250	22 ± 8	1,200 ± 500	3.2	4
**11**	Ψ-2C-O-2	710 ± 30	400 ± 280	99 ± 8	53 ± 4	2,100 ± 1,100	27 ± 8	1,500 ± 900	1.8	3.8
**12**	Ψ-2C-O-3	1,010 ± 30	32 ± 8	200 ± 20	23 ± 10	310 ± 240	17 ± 4	210 ± 140	32	6.6
**13**	Ψ-2C-O-16	910 ± 60	54 ± 4	39 ± 19	49 ± 20	91 ± 31	27 ± 8	500 ± 230	17	9.3
**14**	Ψ-2C-O-21	1,600 ± 100	970 ± 190	32 ± 2	55 ± 14	>10,000		3,500 ± 250	1.6	3.6
**15**	Ψ-2C-O-21.5	1,300 ± 100	1,600 ± 100	740 ± 80	31 ± 5	>10,000		2,800 ± 1,400	0.8	1.8
**16**	Ψ-2C-O-22	2,400 ± 0	600 ± 60	3,400 ± 3,700	41 ± 8	>10,000		2,200 ± 1,100	4	3.7
**17**	Ψ-2C-O-27	2,100 ± 100	8 ± 1	410 ± 160	42 ± 8	>10,000		110 ± 10	280	15
**18**	Ψ-2C-O-35	4,440 ± 70	72 ± 18	260 ± 200	16 ± 2	300 ± 230	20 ± 13	510 ± 140	6.1	7.1
4-alkoxy-substituted 2, 6-dimethoxyamphetamines										
**1**	TMA-6	>5,600	270 ± 18	70 ± 32	61 ± 12	150 ± 20	59 ± 3	1,800 ± 1,000	>21	6.7
**19**	Ψ-MEM	>5,600	490 ± 20	650 ± 210	54 ± 5	260 ± 160	28 ± 11	5,100 ± 1,900	>11	10
**20**	Ψ-MMALM	>5,600	52 ± 10	58 ± 28	68 ± 9	93 ± 26	33 ± 11	290 ± 90	>108	5.6
**21**	Ψ-MALM	>5,600	75 ± 11	180 ± 70	66 ± 6	55 ± 24	69 ± 19	780 ± 330	>75	10
**22**	Ψ-MFEM	>5,600	1,400 ± 300	1,300 ± 90	37 ± 2	>10,000		10,000 ± 4,000	>4	7.1
**23**	Ψ-MDFEM	>5,600	950 ± 240	1,100 ± 400	34 ± 7	>10,000		9,500 ± 1,100	>6	10
**24**	Ψ-MTFEM	>5,600	570 ± 80	1,100 ± 400	33 ± 6	>10,000		5,500 ± 3,000	>10	9.6
**25**	Ψ-MBnM	>5,600	21 ± 2	430 ± 150	39 ± 6	>10,000		270 ± 50	>27	13
**26**	Ψ-DODFMO	5,100 ± 400	84 ± 23	300 ± 100	54 ± 4	160 ± 50	58 ± 23	1,800 ± 1,200	61	21
References substances										
	2C-B^a^	311 ± 46	6.9 ± 1.8	2.1 ± 0.8	92 ± 8	75 ± 14	52 ± 26	43 ± 4	45	6.2
	LSD^a^	1.5 ± 0.4	5.3 ± 3.4	44 ± 14	73 ± 2	>10,000		14 ± 3	0.28	2.6

*K*_i_ and EC_50_ values are given as nM (mean ± SD); activation efficacy (E_max_) is given as percentage of maximum ±SD.

^a^ Data taken from Luethi et al. (Luethi et al., 2018).

#### 3.1.2 5-HT_2A_ receptor

Binding affinities and activation potencies at the 5-HT_2A_ receptor are listed in Table 1. At the 5-HT_2A_ receptor, most phenethylamine derivatives exhibited binding affinity in the submicromolar range, with Ψ-2C-O-16 (**13**), Ψ-2C-O-3 (**12**), and Ψ-2C-O-27 (**17**) showing the highest affinity at this receptor (*K*_i_ = 8–54 nM). The fluorine-containing ethoxy derivatives **14**, **15**, and **16** exhibited a substantially lower binding affinity at the receptor (*K*_i_ = 600–1,600 nM). The difluoromethoxy derivative Ψ-2C-O-35 (**18**) bound the receptor with similar affinity as Ψ-2C-O-16 (**13**), Ψ-2C-O-3 (**12**), and Ψ-2C-O-27 (**17**) (*K*_i_ = 72 nM). The nonfluorinated amphetamine counterparts exhibited submicromolar affinity at the 5-HT_2A_ receptor (*K*_i_ = 21–490 nM), with Ψ-MALM (**21**), Ψ-MMALM (**20**), and Ψ-MBnM (**25**) showing the highest affinities at the receptor. The fluorinated ethoxy derivatives **22**, **23**, and **24** exhibited lower affinities at the 5-HT_2A_ receptor (*K*_i_ = 570–1,400 nM), whereas the difluoromethoxy compound Ψ-DODFMO (**26**, *K*_i_ = 84 nM) was among the compounds with the highest affinities. The phenethylamine derivatives (structures **10**–**18**) activated the 5-HT_2A_ receptor as partial agonists with EC_50_ values ranging from 32 to 3,400 nM and activation efficacies of 16%–84%. The amphetamine counterparts (structures **1, 19**–**26**) were partial agonists at the 5-HT_2A_ receptor with EC_50_ values ranging from 58 to 1,300 nM and activation efficacies of 33%–68%.

#### 3.1.3 5-HT_2B_ receptor

Activation potency at the 5-HT_2B_ receptor is shown in Table 1. Among the phenethylamines, only Ψ-2C-O-1 (**10**), Ψ-2C-O-2 (**11**), Ψ-2C-O-3 (**12**), Ψ-2C-O-16 (**13**), and Ψ-2C-O-35 (**18**), activated the 5-HT_2B_ receptor with EC_50_ values ranging from 91 to 2,100 nM and activation efficacies of 17%–27%. Similarly, the corresponding amphetamine counterparts of the active phenethylamine derivatives, namely TMA-6 (**1**), Ψ-MEM (**19**), Ψ-MMALM (**20**), Ψ-MALM (**21**), and Ψ-DODFMO (**26**) activated the 5-HT_2B_ receptor as partial agonists with EC_50_ values ranging from 55 to 260 nM and activation efficacies of 28%–69%. The remaining phenethylamine and amphetamine derivatives did not activate the receptor in the examined concentration range (EC_50_ > 10 μM).

#### 3.1.4 5-HT_2C_ receptor

5-HT_2C_ receptor affinities are listed in Table 1. Ψ-2C-O-3 (**12**), Ψ-2C-O-16 (**13**), Ψ-2C-O-27 (**17**), and Ψ-2C-O-35 (**18**) exhibited submicromolar binding affinity at the 5-HT_2C_ receptor (*K*_i_ = 110–510 nM). The remaining phenethylamine derivatives (**10, 11, 14**–**16**) bound to the receptor with micromolar affinity (*K*_i_ = 1,200–3,500 nM). Among the amphetamines, Ψ-MMALM (**20**), Ψ-MALM (**21**), and Ψ-MBnM (**25**) bound to the 5-HT_2C_ receptor at submicromolar concentrations (*K*_i_ = 270–780 nM). The remaining amphetamine derivatives displayed lower binding affinities (*K*_i_ = 1,800–10,000 nM).

### 3.2 Interactions with non-serotonergic receptors and monoamine transporters

Interactions with non-serotonergic receptors and monoamine transporters are shown in Table 2. No relevant interactions were observed for the phenethylamine and amphetamine series at the D_2_ receptor (*K*_i_ > 6 µM). Ψ-2C-O-27 (**17**) and Ψ-MBnM (**25**) exhibited micromolar binding affinity at the DAT (*K*_i_ = 1.3 and 1.8 µM, respectively) and NET (*K*_i_ = 6.2 and 7.1 µM, respectively), whereas no binding to SERT was observed (*K*_i_ < 7.5 µM). Ψ-2C-O-3 (**12**), Ψ-MMALM (**20**), Ψ-MDFEM (**23**), and Ψ-MTFEM (**24**) bound to DAT in the range of 5.4–7.8 µM. None of the test substances displayed >50% monoamine uptake inhibition at any transporter (data not shown). The phenethylamine derivatives (**10**–**18**) exhibited high affinities at the rat TAAR1 (*K*_i_ = 1.6–57 nM) and lower affinity at mouse TAAR1 (*K*_i_ = 120–680 nM). With the exception of Ψ-MBnM (**25**), the corresponding amphetamine counterparts (**1, 19**–**24, 26**) bound with decreased affinity to the rat TAAR1 (*K*_i_ = 6–180 nM) and the mouse TAAR1 (*K*_i_ = 190–860 nM). At the human TAAR1, Ψ-2C-O-2 (**11**), Ψ-2C-O-3 (**12**), Ψ-2C-O-16 (**13**), Ψ-2C-O-21 (**14**), Ψ-2C-O-21.5 (**15**), and Ψ-2C-O-22 (**16**) activated the receptor at submicromolar concentrations ranging from 34 to 230 nM, whereas Ψ-2C-O-1 (**10**) and Ψ-2C-O-27 (**17**) displayed low micromolar activation potency (EC_50_ of 2.3 and 3.0 µM, respectively). Ψ-2C-O-35 did not activate the human TAAR1 at investigated concentrations (EC_50_ > 30 µM). The amphetamine derivatives Ψ-MEM (**19**), Ψ-MMALM (**20**), Ψ-MFEM (**22**), Ψ-MDFEM (**23**), and Ψ-MTFEM (**24**) activated the human TAAR1 receptor with EC_50_ values ranging from 850 to 2,500 nM, while the remaining derivatives showed no relevant interactions with the receptor (EC_50_ > 30 µM). Ψ-2C-O-3 (**12**) and Ψ-2C-O-16 (**13**) were the only substances to exhibit submicromolar affinity binding to the α_1A_ receptor (*K*_i_ = 670–990 nM). The remaining phenethylamine (**10,14**–**18**) and amphetamine derivatives (**1,19**–**26**) exhibited less potent binding affinity (*K*_i_ > 1,400 µM). The phenethylamine derivatives (**10**–**18**) bound to the α_2A_ receptor with affinities in the range of 280–1,500 nM, while the amphetamine counterparts (**1,19**–**26**) bound with low micromolar affinities (*K*_i_ = 1,400–4,100 nM).

**TABLE 2:** Monoamine receptor and transporter binding affinities of 4-alkoxy-substituted 2,6-dimethoxyphenethylamines and amphetamines.

		hTAAR1	rTAAR1	mTAAR1	hα_1A_	hα_2A_	hD_2_	hNET	hDAT	hSERT
		EC_50_ ± SD [nM]	*K*_i_ ± SD [nM]	*K*_i_ ± SD [nM]	*K*_i_ ± SD [nM]	*K*_i_ ± SD [nM]	*K*_i_ ± SD [nM]	*K*_i_ ± SD [nM]	*K*_i_ ± SD [nM]	*K*_i_ ± SD [nM]
			[^3^H]RO5166017	[^3^H]RO5166017	[^3^H]prazosin	[^3^H]rauwolscine	[^3^H]spiperone	*N*-methyl-[^3^H]nisoxetine	[^3^H]WIN35,428	[^3^H]citalopram
4-alkoxy-substituted 2,6-dimethoxyphenethylamines										
**10**	Ψ-2C-O-1	2,300 ± 1,900	57 ± 12	590 ± 20	>8,800	780 ± 70	>6,000	>8,800	>8,500	>7,500
**11**	Ψ-2C-O-2	230 ± 170	1.6 ± 5	230 ± 20	3,300 ± 300	670 ± 60	>6,000	>8,800	>8,500	>7,500
**12**	Ψ-2C-O-3	100 ± 60	6.4 ± 1.1	120 ± 20	990 ± 20	280 ± 30	>6,000	>8,800	7,800 ± 500	>7,500
**13**	Ψ-2C-O-16	34 ± 15	12 ± 0	150 ± 20	670 ± 60	310 ± 20	>6,000	>8,800	>8,500	>7,500
**14**	Ψ-2C-O-21	80 ± 43	19 ± 1	320 ± 30	4,600 ± 200	1,500 ± 7,200	>6,000	>8,800	>8,500	>7,500
**15**	Ψ-2C-O-21.5	41 ± 9	14 ± 3	220 ± 40	2,000 ± 200	1,000 ± 200	>6,000	>8,800	>8,500	>7,500
**16**	Ψ-2C-O-22	47 ± 22	21 ± 2	210 ± 40	2,000 ± 400	1,500 ± 100	>6,000	>8,800	>8,500	>7,500
**17**	Ψ-2C-O-27	3,000 ± 500	3.1 ± 0.2	610 ± 6	3,600 ± 300	410 ± 20	>6,000	6,200 ± 800	1,300 ± 100	>7,500
**18**	Ψ-2C-O-35	>30,000	51 ± 4	680 ± 120	2,100 ± 200	450 ± 70	>6,000	>8,800	>8,500	>7,500
2,6-dimethoxyamphetamines										
**1**	TMA-6	>30,000	180 ± 20	850 ± 20	>8,800	3,100 ± 300	>6,000	>8,800	>8,500	>7,500
**19**	Ψ-MEM	2,200 ± 1,900	110 ± 10	400 ± 20	6,010 ± 590	2,200 ± 0	>6,000	>8,800	>8,500	>7,500
**20**	Ψ-MMALM	2,500 ± 300	32 ± 5	250 ± 50	2,200 ± 300	1,400 ± 100	>6,000	>8,800	5,700 ± 300	>7,500
**21**	Ψ-MALM	>30,000	58 ± 11	270 ± 50	1,400 ± 0	1,800 ± 200	>6,000	>8,800	>8,500	>7,500
**22**	Ψ-MFEM	1,900 ± 600	52 ± 18	560 ± 10	6,700 ± 900	4,100 ± 400	>6,000	>8,800	>8,500	>7,500
**23**	Ψ-MDFEM	850 ± 500	66 ± 13	500 ± 70	3,900 ± 400	3,300 ± 400	>6,000	>8,800	5,400 ± 400	>7,500
**24**	Ψ-MTFEM	1,100 ± 600	110 ± 40	410 ± 80	6,100 ± 500	3,800 ± 100	>6,000	>8,800	6,400 ± 700	>7,500
**25**	Ψ-MBnM	>30,000	6.1 ± 0.7	190 ± 10	5,800 ± 400	2,000 ± 0	>6,000	7,100 ± 900	1,800 ± 200	>7,500
**26**	Ψ-DODFMO	>30,000	140 ± 40	860 ± 40	5,900 ± 800	2,300 ± 100	>6,000	>8,800	>8,500	>7,500
References susbtances										
	MDMA^a^		250 ± 10	3,100 ± 700	6,900 ± 1,200	4,600 ± 100	>13,000	>8,700	>8,500	>7,500

Ki and EC50 values are given as nM (mean ± SD); activation efficacy (Emax) is given as percentage of maximum ±SD. ^a^Data taken from Luethi et al. (2019b).

## 4 Discussion

### 4.1 Interactions with serotonergic receptors

#### 4.1.1 5-HT_1A_ receptor binding affinity

The Ψ-2C derivatives exhibited moderate to low binding affinity at the 5-HT_1A_ receptor, with the most potent derivative being Ψ-2C-O-2 (**11**, *K*_i_ = 710 nM). In contrast, none of the amphetamine-based derivatives showed significant affinities at this receptor subtype (*K*_i_ *>* 5 µM). This indicates a disadvantageous effect of α-methylation for 5-HT_1A_ receptor binding, as already observed for other compared series (Kolaczynska et al., 2019; Rickli et al., 2019; Simmler et al., 2013). Among the phenethylamine series, minor extensions of the 4-alkoxy moiety did not affect 5-HT_1A_ binding. However, a benzyloxy or fluorinated substituents at the 4-position of the Ψ-2C derivatives slightly reduced the binding affinity at the receptor. Similar effects were previously observed for 2,4,5-trisubstituted derivatives (Kolaczynska et al., 2019); however, the observed affinities at the 5-HT_1A_ receptor for these compounds were overall lower (e.g., 2C-O-2; **28**: *K*_i_ = 3,600 nM vs. Ψ-2C-O-2; **11**: *K*_i_ = 710 nM).

#### 4.1.2 5-HT_2A_ receptor interactions

Generally, and as observed earlier for many other 5-HT_2A_ ligands with the phenethylamine pharmacophore (Nichols, 2016; Nichols, 2004; Trachsel et al., 2013; Luethi and Liechti, 2018; Glennon et al., 1986; Luethi and Liechti, 2020), an expansion of the critical 4-substituent with lipophilic groups leads to an increase in affinity among the compounds investigated herein (Table 1). This trend can be observed for both the phenethylamine and amphetamine series. The compounds with 4-substituents of largest steric bulkiness, i.e. a 4-benzyloxy group, displayed the highest affinities (Ψ-2C-O-27; **17**: *K*_i_ = 8 nM and Ψ-MBnM; **25**: *K*_i_ = 21 nM), but they had decreased activation potencies and efficacies in comparison to their simplest analogs, the 4-methoxy compounds. Generally, the phenethylamine compounds with an extended 4-substution showed a decreased efficacy in comparison to the parent compound Ψ-2C-O-1 (**10**). Among the amphetamines, this trend was only partially observed, and earlier observations showing generally higher intrinsic activities for racemic amphetamines compared to their 2C phenethylamine congeners (Parrish et al., 2005) were not clearly confirmed. All investigated compounds behaved as partial agonists (Table 1, activation efficacies = 23–84%).

Previously, we reported on the effects of similar 4-position modifications among 4-alkoxy-2,5-dimethoxy (2C-O and MRM derivatives) and 4-alkoxy-3,5-dimethoxy (scalines and 3C-scalines) derivatives (Kolaczynska et al., 2019; Kolaczynska et al., 2022) (Figures 3, 4). Overall, 5-HT_2A_ receptor binding of 2C-O and MRM derivatives was similar to their individual pseudo counterparts (<2-fold difference). However, TMA-6 (**1**), Ψ-2C-O-16 (**13**), and Ψ-MALM (**21**) displayed a 2–5-fold higher affinity compared to TMA-2 (**2**), 2C-O-16 (**30**), and MALM (**47**), respectively. Compared to their Ψ derivative counterparts, the 2C-O and MRM derivatives exhibited similar or higher activation potency and higher activation efficacy (47%–95% vs. 33%–68%) at the 5-HT_2A_ receptor (Kolaczynska et al., 2019). MMALM (**46**) and MALM (**47**) even activated the receptor as full agonists, while their pseudo counterparts, Ψ-MMALM (**20**) and Ψ-MALM (**21**), respectively, were partial agonists (Kolaczynska et al., 2019). Scalines and 3C-scalines were partial to full 5-HT_2A_ receptor agonists with decreased *in vitro* potency compared to their 2,4,5- and 2,4,6-trisubsituted counterparts (Kolaczynska et al., 2022).

Among the compounds investigated herein, introduction of fluorine atoms onto the terminal position of a 4-ethoxy group lowered affinities in comparison to the non-fluorinated analog Ψ-2C-O-2. However, by increasing the fluorine substituents from F_1_ to F_3_ the affinities increased by trend. The maximum affinity loss by fluorine introduction was around three-fold. This adds additional evidence for the unique physico-chemical properties of fluorine substituents. While a single fluorine atom is considered to have a *Van der Waals* radius with only some 20% increase over a hydrogen atom (with some models stating it is close to an oxygen), a trifluoromethyl group is considerably larger than its comparator, a methyl group, and is being close to the steric bulkiness of an isopropyl, a *tert*-butyl, or even a phenyl group, depending on the calculating model (Kirk, 2006; Hagmann, 2008; Ojima, 2009). Not only steric changes, but also properties such as lipophilicity—a fluorine attached to an alkyl chain can even decrease its lipophilicity (detrimental to 5-HT_2A_ affinity)— a changed dipole moment, the possibility to form so-called multipolar interactions (e.g. C–F···C=O), or the ability of a fluorine to act as an hydrogen acceptor (although weaker than an oxygen) can lead to unpredictable advantageous or disadvantageous properties. Generally, introduction of fluorine atoms at critical positions can dramatically change a molecule’s biological and chemical properties, which sets fluorine as a valuable tool for medicinal chemistry. Among earlier investigated phenethylamines, a similar trend, i.e. an initial decrease and subsequent progressive increase of 5-HT_2A_ receptor affinities was observed when introducing F_1_ to F_3_ into the terminal position of a 4-ethoxy group of 3,5-dimethoxy (scalines and 3C-scalines) as well as 2,5-dimethoxy (2C-O and MRM series) derivatives (Trachsel et al., 2013; Kolaczynska et al., 2019; Kolaczynska et al., 2022). Notably, at least from the scaline series, the corresponding human doses for psychedelic effects are known, and their order of ranking is corresponding to their affinities: an increased affinity lead to higher human potency (Trachsel et al., 2013; Kolaczynska et al., 2022; Luethi and Liechti, 2018). In contrast, although no complete comparable set was investigated, among 4-thio-substituted 2,5-dimethoxyphenethylamines, a 4-(2,2-difluoroethyl)thio and a 4-(2,2,2-trifluoroethyl)thio group lead to compounds with nearly identical affinities, being among the most potent 5-HT_2A_ ligands with smaller 4-substituents from that series (Luethi et al., 2018); Fluorination could alter enzymatic degradation of a substance, thereby potentially affecting its clinical potency (Clark et al., 1965). However, from human data it is known that nearly no difference in active dose ranges can be observed among the fluorine-free compound (2C-T-2), the monofluoro analog (2C-T-21), and the difluoro analog (2C-T-21.5) (Trachsel et al., 2013; Shulgin and Shulgin, 1991). This further demonstrates how unpredictable the effects of fluorine introduction can be. Fluorination of the 4-ethoxy group had detrimental effects on activation potencies as well as on efficacies on both phenethylamine and amphetamine series (Table 1), with the exception for Ψ-2C-O-21 (**14**) that showed a 3-fold increase in activation potency over its non-fluorinated counterpart Ψ-2C-O-2 (**11**). For 4-alkoxy 2,5-dimethoxy compounds (2C-O and MRM derivatives) and 4-alkoxy 3,5-dimethoxy compounds (scalines and 3C-scalines), no clear trends were observed for fluorination regarding 5-HT_2A_ activation potencies and efficacies (Kolaczynska et al., 2019; Kolaczynska et al., 2022). In contrast to the fluorination effects on a 4-ethoxy substituent as outlined before, a somewhat different effect was observed for the pairs Ψ-2C-O-1 (**10**)/Ψ-2C-O-35 (**18**) and TMA-6 (**1**)/Ψ-DODFMO (**26**). Herein, the 5-HT_2A_ affinities were increased 3–4-fold (Table 1). A similar effect of fluorination of the 4-methoxy substituent could be observed within a series of scalines in our earlier work (Kolaczynska et al., 2022), wherein the receptor affinities as well as human potencies were significantly increased from mescaline to difluoromescaline and trifluoromescaline (*K*_i_ values: 3-fold and 33-fold increase, respectively; human potencies: 4-fold and > 9-fold increase, respectively). In contrast to the effects on affinity, fluorination of the 4-methoxy group had detrimental effects on activation potencies as well as on efficacies on both phenethylamine and amphetamine series investigated herein (Table 1). This is in contrast to the observed increase in activation potencies and efficacies for progressive fluorination of the 4-methoxy group of mescaline (Kolaczynska et al., 2022). Interestingly, the effect of fluorination on 5-HT_2A_ receptor efficacy was much more pronounced for Ψ-2C-O-35 (**18**; efficacy of 16%) compared to its 3C analog Ψ-DODFMO (**26**; efficacy of 54%). The reason for this is, however, unclear and the relatively potent human psychoactive properties (Trachsel et al., 2013) do not reflect their low efficacies. This could indicate that signal transduction pathways other than those investigated here are relevant.

Taken together, when comparing the 5-HT_2A_ receptor interaction data of the three series (i.e., the 4-alkoxy substituted 3,5-dimethoxy, 2,5-dimethoxy and 2,6-dimethoxy series), it appears that the 2,5-dimethoxy substitution pattern fits best for 4-alkoxy substituents. This is partly reflected by human potencies known for corresponding derivatives. Furthermore, a careful comparison of other investigated 4-substituents revealed that it cannot generally be concluded that one distinct class always bears the most active compound among the three substitution pattern classes (Trachsel et al., 2013).

Compared to their 2,4,5-trisubstituted counterparts, 2,4,6-trisubtituted derivatives are generally more difficult to synthesize (Trachsel, 2012) and only limited information on them is currently available. Nevertheless, a few derivatives from the pseudo series have previously been evaluated in terms of *in vitro* or *in vivo* potency (Chambers et al., 2002; Glennon and Young, 1982; Shulgin and Shulgin, 1991; Parker et al., 2008; Glennon et al., 1981; Nichols et al., 1991). Compared to the potent psychedelic amphetamine DOM (**5**; 3–10 mg, 14–20 h), the 2,4,6-substituted Ψ-DOM (**4**; 15–25 mg, 6–8 h) retains psychoactivity in humans but is less potent (Figure 1). A similar pattern can be observed for the 2,4,5-trisubstituted amphetamine TMA-2 (**2**; 20–40 mg, 8–12 h), and its pseudo counterpart TMA-6 (**1**; 25–50 mg, 12–16 h) (Shulgin and Shulgin, 1991). Metabolism of trialkoxylated phenethylamines with different aryl-substitution patterns seems to be a critical factor that accounts for differences in potency (Clark et al., 1965). The potency differences of the aforementioned compounds have also been observed in animal drug discrimination models with comparable outcome (Glennon et al., 1981, Glennon and Young 1982, Nichols et al., 1991, Chambers et al., 2002, Parker et al., 2008). From limited data available for the third comparators of previously known compounds, a comparison with anecdotal evaluation in human did so far not allow a conclusion, as active levels are only known for 2C-T-4 (**8**; 8–20 mg, 12–18 h), while they remain unexplored for Ψ-2C-T-4 (**7**; >12 mg) (Shulgin and Shulgin, 1991).

It is important to note that for phenethylamine ligands bearing a 3,4,5-trisubstitution pattern, a different orientation (i.e., out-of-plane conformation) of the 3,5-dimethoxy substituents towards the 5-HT_2A_ receptor was proposed when compared to their 2,4,5 congeners (Monte et al., 1997). Furthermore, a site-directed mutagenesis study gave further evidence for different binding orientation between the two series (Trachsel et al., 2013). There are indications of a different binding orientation for 2,4,6-trisubstituted phenethylamines as well (Chambers et al., 2002; Monte et al., 1996) Hitherto, the effect of various binding orientation of 4-substituted dimethoxy phenethylamines to receptor interaction and its subsequent signaling pathways remains largely unclear and it is likely that this significantly contributes to the differences observed between the phenethylamines with the three substitution patterns.

The 5-HT_2A_ receptor is coupled to different intracellular signaling cascades. Canonically, the receptor is coupled to the Gα_q_ protein, which upon receptor activation activates phospholipase C (PLC), resulting in hydrolysis of membrane phosphoinositides to inositol triphosphate (IP_3_) and diacylglycerol (DAG) (Pottie and Stove, 2022). This leads to the mobilization of Ca^2+^ and activation of protein kinase C (PKC) further downstream. In addition, activation of phospholipase A_2_ (PLA_2_) with subsequent arachidonic acid release and β-arrestin recruitment are linked to 5-HT_2A_ receptor activation (Pottie and Stove, 2022). Psychedelics may activate different signaling pathways with different potencies, resulting in biased signaling (Pottie et al., 2023). It is important to note that 5-HT_2_ receptor activation potencies of this study were assessed by Ca^2+^ mobilization, which is a poor predictor of clinical potency (Luethi and Liechti, 2018). To gain more precise insight into 5-HT_2A_ receptor interactions of Ψ derivatives, additional readouts of the same signaling pathway (e.g., IP_1_/IP_3_ accumulation) could be evaluated, or G protein dissociation could be directly measured using bioluminescence resonance energy transfer (BRET) assays (Bonniwell et al., 2025).

#### 4.1.3 5-HT_2B_ receptor activation potency and efficacy

While some of the smaller 4-substituents in both the phenethylamine and amphetamine compounds investigated lead to submicromolar activation potencies, derivatives containing a benzyl group in their substituent (Ψ-2C-O-27 (**17**) and Ψ-MBnM (**25**)) did not activate the human 5-HT_2B_ receptor (EC_50_ > 10,000 nM), which is in line with earlier observations on other aryl substitution patterns, wherein large 4-substituents lead to low activation potencies (Luethi et al., 2019a). Surprisingly, either a mono-, di-, or trifluorination at the terminal position of a 4-ethoxy substituent lead to a complete loss of activation in contrast to the non-fluorinated analogs Ψ-2C-O-2 (**11**) and Ψ-MEM (**19**), revealing the sensitivity of this receptor subtype towards such modifications. However, the 4-(2,2-difluoromethoxy)-substituted derivatives Ψ-2C-O-35 (**18**) and Ψ-DODFMO (**26**) essentially retained the abilities of 5-HT_2B_ receptor activation, with identical efficacies, in comparison to their non-fluorinated analogs Ψ-2C-O-1 (**10**) and TMA-6 (**1**). Among the derivatives that activated the 5-HT_2B_ receptor, each amphetamine derivative showed higher activation potency (EC_50_ = 55–260 nM vs. 91–2,100 nM) and efficacy (28%–69% vs. 17%–27%) compared to its phenethylamine counterpart; the most distinct (i.e., 8-fold) difference in activation potency was observed for the comparators Ψ-2C-O-2 (**10**) and Ψ-MEM (**19**). Currently there is no obvious explanation for this effect. Furthermore, both smaller as well as larger 4-alkoxy substituents lead to higher activation potencies (i.e., a methoxy, an allyloxy, or even a methallyloxy group) at the 5-HT_2B_ receptor compared to the 4-ethoxy-substituted substances. This suggests either critical steric bulkiness, conformational issues, or other physicochemical properties of the 4-ethoxy group towards this receptor subtype. Some distinct effects of fluorination of a 4-ethoxy substituent onto 5-HT_2B_ activation potencies and efficacies was also shown for 3,4-5- and especially 2,4,5-trisubstituted phenethylamines and amphetamines (Kolaczynska et al., 2019; Kolaczynska et al., 2022).

Chronic activation of the 5-HT_2B_ receptor by stimulants has been linked to adverse effects including cardiomyopathy (Fitzgerald et al., 2000; Rothman et al., 2000; Luethi et al., 2021). Most Ψ derivatives were weak to moderate partial agonists at the receptor, which still potentially could put regular users of these compounds at a risk of developing the aforementioned cardio-related adverse effects (Luethi et al., 2021). This is a familiar potential issue among the 2,4,5-trisubstituted counterparts which are partial to full agonists at the 5-HT_2B_ receptor (Kolaczynska et al., 2019) and may carry a potential risk of cardiomyopathy for frequent drug users. However, it currently remains unclear whether psychedelics that potently activate the 5-HT_2B_ receptor could mediate 5-HT_2B_ receptor-mediated adverse effects.

#### 4.1.4 5-HT_2C_ receptor binding affinity

Similar to the trends observed at the 5-HT_2A_ receptor, the binding affinity at the 5-HT_2C_ receptor was decreased by 4-ethoxy vs. 4-methoxy substitution; however, further side chain prolongation progressively increased the 5-HT_2C_ receptor affinity. Fluorination enhanced the *K*_i_ only for Ψ-2C-O-35 (**18**) compared to Ψ-2C-O-2 (**11**) and for none of the amphetamine derivatives compared to TMA-6 (**1**). In contrast to the binding data from the 5-HT_2A_ receptor assays, all amphetamines showed somewhat lower affinities than their direct phenethylamine counterparts at the 5-HT_2C_ receptors. At the 5-HT_2A_ receptor this trend was not obvious.

#### 4.1.5 5-HT receptor selectivity (2A vs. 1A and 2A vs. 2C)

Among the investigated 5-HT receptor subtypes, most phenethylamine and amphetamine derivatives exhibited modest binding preference to the 5-HT_2A_ over the 5-HT_2C_ or 5-HT_1A_ receptor. This is in line with previous reports on similar psychedelic phenethylamine and amphetamine derivatives (Luethi et al., 2018; Kolaczynska et al., 2019; Kolaczynska et al., 2022; Glennon et al., 1986; Glennon et al., 1980; Nichols et al., 1994) (Table 1). Ψ-2C-O-2 (**11**) and Ψ-2C-O-21 (**14**) were equipotent agonists at the 5-HT_2A_ and 5-HT_1A_ receptors (<2-fold preference). Ψ-2C-O-21.5 (**15**) was an equipotent agonist at the 5-HT_2A_, 5-HT_2C_, and 5-HT_1A_ receptor. Extending the 4-alkoxy chain of the phenethylamines augmented the 5-HT_2A_ vs. 5-HT_1A_ binding preference up to 280-fold as observed for Ψ-2C-O-27 (**17**). Fluorination of the 4-substituent did not substantially affect 5-HT_2A_ vs. 5-HT_1A_ selectivity. In terms of the selectivity for the 5-HT_2A_ vs. 5-HT_2C_ receptor, the binding ratios ranged from 1.8 to 15 for the phenethylamines and from 6.7 to 21 for the amphetamine-based derivatives. Compared to the 2,4,5-trisubstituted derivatives, the 5-HT_2A_ vs. 5-HT_2C_ receptor selectivity ratio was analogous for the phenethylamines and the amphetamine based derivatives (Kolaczynska et al., 2019). *In vitro* binding affinities assessed for these derivatives at the 5-HT_2A_ and 5-HT_2C_ receptors may be one of several factors (in addition to, e.g., intrinsic receptor activities, metabolism, bioavailability, and blood brain barrier penetration) that predict their potential clinical potency in humans (Luethi and Liechti, 2018).

### 4.2 Non-serotonergic receptor and monoamine transporter interaction

The Ψ derivatives did not interact with the D_2_ receptor and showed mostly no significant affinity at the monoamine uptake transporters. This is in line with studies of related substances, including 2C-O and MRM derivatives, which share the same 4-position moieties (Luethi et al., 2018; Rickli et al., 2015; Kolaczynska et al., 2019; Kolaczynska et al., 2022). Noteworthy exceptions included Ψ-2C-O-27 (**17**) and Ψ-MBnM (**25**), which exhibited moderate affinity (*K*_i_ = 1.3–1.8 μM) at DAT. Both derivatives possess a benzyloxy group as 4-substituent, which has previously been shown to increase binding to and likely also cause inhibition of DAT for the related derivative 2C-O-27 (Kolaczynska et al., 2019). Furthermore, various 4-aryl-substituted 2C derivatives interact with monoamine transporters (Luethi et al., 2019a). Ψ-2C-O-3 (**12**), Ψ-MMALM (**20**), Ψ-MDFEM (**23**), and Ψ-MTFEM (**24**) exhibited weak affinity at DAT (*K*_i_ = 5.4–7.8 μM) as well, suggesting that bulky 4-substituents of Ψ derivatives are favorable for DAT binding.

Generally, most investigated derivatives bound with slight preference to the α_2A_ receptor (*K*_i_ = 280–4,100 nM) vs. the α_1A_ receptor (*K*_i_ = 670–6,700 nM), as previously observed for other substituted phenethylamines (Luethi et al., 2018; Rickli et al., 2015; Kolaczynska et al., 2019; Kolaczynska et al., 2022). Ψ-2C-O-16 (**13**) and Ψ-2C-O-3 (**12**) exhibited the highest affinity at the α_2A_ and α_1A_ receptor, respectively. Extension of the carbon chain moiety and 4-benzyloxy substitution in both phenethylamine and amphetamine derivatives (i.e., Ψ-2C-O-1 (**10**), Ψ-2C-O-2 (**11**), Ψ-2C-O-3 (**12**), Ψ-2C-O-16 (**13**), TMA-6 (**1**), Ψ-MEM (**19**), Ψ-MMALM (**20**), and Ψ-MALM (**21**)) enhanced the binding affinity at both adrenoceptors. In contrast, fluorination (i.e., Ψ-2C-O-21 (**14**), Ψ-2C-O-21.5 (**15**), Ψ-2C-O-22 (**16**), Ψ-MFEM (**22**), Ψ-MDFEM (**23**), and Ψ-MTFEM (**24**)) did not enhance binding affinity. Similar findings were observed with the 2,4,5-trisubstituted derivatives containing the same 4-position moieties (Kolaczynska et al., 2019). However, in general these derivatives exhibited less potent binding affinity at both adrenoceptors.

The majority of the derivatives exhibited greater affinity to the rat TAAR1 (*K*_i_ = 1.6–180 nM) vs. mouse TAAR1 (*K*_i_ = 120–860 nM). The observed rank order at the receptor (rat > mouse > human TAAR1) is in agreement with previous reports investigating other substituted phenethylamines and amphetamines containing similar structural modifications (Luethi et al., 2019a; Luethi et al., 2018; Kolaczynska et al., 2019; Kolaczynska et al., 2022). The overall binding affinity at the rat and mouse TAAR1 for 2,4,5-trisubstituted derivatives was several orders of magnitude lower (e.g., *K*_i_ of Ψ-2C-O-2 (**11**) = 1.6 nM vs. *K*_i_ of 2C-O-2 (**28**) = 670 nM). 4-Ethoxy, 4-methallyloxy, and 4-allyloxyphenethylamines (Ψ-2C-O-2 (**11**), Ψ-2C-O-3 (**12**), and Ψ-2C-O-16 (**13**)) showed higher activation potency at human TAAR1 compared to derivatives with a short (i.e., 4-methoxy) or bulky (i.e., 4-benzyloxy) substituent. Fluorination of the 4-ethoxy substituent (Ψ-2C-O-21 (**14**), Ψ-2C-O-21.5 (**15**), and Ψ-2C-O-22 (**16**)) further increased the potency at human TAAR1. All remaining derivatives interacted with the receptor in the micromolar range (EC_50_ = 1,100–2,500 nM) or did not activate the receptor (EC_50_ > 30,000 nM). In general, the presence of an α-methyl group (**1,19–26**) reduced the binding affinity observed for comparable derivatives lacking the group (**10–18**) at both adrenoceptors and at TAAR1.

## 5 Conclusion

We examined several monoamine receptor and transporter interaction properties of 4-alkoxy-2,6-dimethoxy-substituted phenethylamine and amphetamine derivatives *in vitro*. In general, these so-called Ψ derivatives interacted with receptors in a way that is common among known psychedelics. Various of the Ψ derivatives investigated herein displayed a promising 5-HT_2A_ receptor profile, which suggests psychoactivity in humans. Clearly, potency, duration of action and effects in humans may differ from their 2,4,5-trisubstituted counterparts due to parameters such as different pharmacokinetics or varying efficacies in the subsequent signaling pathways. Among the investigated compounds, from at least two compounds some anecdotal information is available. As such, the phenethylamine Ψ-2C-O-35 (**18**) and its amphetamine counterpart Ψ-DODFMO (**26**) have shown some psychedelic effects at initial doses of 17 mg and 2 × 5 mg, respectively, with surprisingly long-lasting effects of 18–20 h (Trachsel et al., 2013). This not only shows that some of these novel compounds can be relatively potent in human, but also reveals that the introduction of two fluorine atoms onto the 4-methoxy group of Ψ-2C-O-1 (**10**; an inactive compound at doses up to 300 mg), greatly changes its biological activity. The relatively high human potency seems surprising when relating to its very low activation efficacy (16%). However, calcium mobilization is a readout of only one of several signaling pathways and is generally a weak predictor of the clinical potency of a 5-HT_2A_ receptor agonist (Luethi and Liechti, 2018).

Overall, extension of the 4-alkyloxy moiety increased receptor binding affinity at 5-HT 2A and 2C receptors, while fluorination of the 4-alkoxy substituent had mixed effect. Furthermore, α-methylation of Ψ-2C derivatives had little effect on the receptor binding and activation properties. Predicted purely on 5-HT_2A_ receptor interaction profiles obtained herein, the phenethylamine-based compounds Ψ-2C-O-3 (**12**) and Ψ-2C-O-16 (**16**), as well as the amphetamine-based derivatives Ψ-MALM (**21**), and Ψ-MMALM (**20**) would be assumed to be some of the most potent compounds in human investigated herein, and such Ψ derivatives may prove to be interesting drug candidates as psychotherapeutics for the treatment of neurological disorders. Lead optimization and potential *in vivo* studies with Ψ derivatives in the future should therefore focus on these four substances.

## Data Availability

The original contributions presented in the study are included in the article/supplementary material, further inquiries can be directed to the corresponding author.
